# Shape and Size Adaptations of Planthoppers Along an Altitudinal Gradient on Mount Wilhelm (Papua New Guinea)

**DOI:** 10.1002/ece3.71479

**Published:** 2025-06-17

**Authors:** De Filippo Elsa, Soulier‐Perkins Adeline, Cornette Raphaël, Guilbert Eric

**Affiliations:** ^1^ UMR7179 MECADEV Muséum National D'histoire Naturelle Paris France; ^2^ UMR7205 ISYEB Muséum National D'histoire Naturelle Paris France

**Keywords:** elevational gradient, environmental filtering, Fulgoromorpha, morphometric analysis, niche conservatism, phylogeny

## Abstract

Mountains are an ideal context to study species community and adaptation in relation to environmental changes such as temperature and vegetation profile. Such changes produce different ecological niches that can be a source of local adaptations in the communities, like body size varying with elevation, for example. In this context, planthoppers (Insects, Hempitera, Fulgoromorpha) community and their species traits were studied along an altitudinal gradient in Mount Wilhelm (Papua New Guinea) to test niche and morphological trait conservatism in relation to environmental filtering. Niche conservatism is significant at high altitude for Cixiidae, and forewing shape is conserved. This is not the case for Achilidae and Derbidae, for which forewing shape is not conserved and forewing relative length increases in size with altitude. These variations in size and shape translate an adaptation of Achilidae and Derbidae to high altitudes; while closely related species of Cixiidae tend to keep the same ecological niche, and then, forewing size and shape are maintained.

## Graphical abstract

Mountains are an ideal context to study species community and adaptation in relation to environmental changes such as temperature. Cixidae show niche conservatism at high altitude, with conserved forewing size and shape. Conversely, size and shape variations of forewings of Achilidae and Derbidae traduce an adaptation to high altitudes.

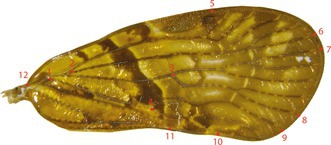

## 1 Introduction

Competition theory predicts that if two species have identical niches, either one species will exclude the other, causing spatial and/or temporal partitioning (Diamond 1975's checkerboard distribution, Gotelli and McCabe 2002; Chao et al. 2005); or selective pressure will eventually result in character displacement (Dayan and Simberloff 2005). However, the physical environment also imposes ecological and evolutionary constraints that create an ‘ecological filter’ such that species with similar ecological requirements are found in similar environments, a pattern referred to as spatial niche clustering or niche conservatism (Keddy 1992; Myers and Harms 2009). Webb et al. (2002) suggested a method to combine phylogenetic and ecological approaches to discriminate historical contingencies from environmental filtering. Approaches combining phylogeny, traits, and communities help in examining the phylogenetic structure of community assemblages; exploring the phylogenetic basis of community niche structure; and adding community context to studies of trait evolution (Webb et al. 2002). Such approaches were developed along altitudinal gradients, as the phylogenetic structure of natural communities may illuminate the processes governing the assembly and coexistence of species in ecological communities (Hardy and Senterre 2007). Altitudinal gradients are an ideal context to study adaptations linked to environmental conditions (Körner 2007). Indeed, along an altitudinal gradient, continuous or discontinuous variations, particularly in temperature and vegetation, produce different ecological niches that can be source of local adaptations in the communities inhabiting them. These variations occur on a relatively small geographical scale and can cover a wide range of conditions (Körner 2007), which explains their interest in studies of morphological or physiological adaptations linked to the environment (Sanborn et al. 2011). Bergmann's rule is the main morphological trait being studied in ectotherm species, and several studies show that body size vary with elevation gradient, according to temperature and precipitation (Kang et al. 2023; Xing et al. 2018). It has also been shown that temperature is a main driver of ground beetles species tolerance at high altitudes (García‐Robledo et al. 2016), but patterns of morphology are not obvious along altitudinal gradient among ground beetles (Maveety and Browne 2014). Such a rule is not obvious according to the species studied, as it is the case among Hymenoptera (Kang et al. 2023). Other traits such as the thermal melanism on ectothermal vertebrates was investigated (Clusella‐Trullas et al. 2007) and show variations among altitudinal gradients. Wing size and shape in relation with environmental parameters were also debated among insects as a proxy of dispersal ability, leading to inconsistent patterns with elevation (Malmqvist 2000; McCulloch and Waters 2017; Levy and Nufio 2015; Rendoll‐Cárcamo et al. 2023). However, most studies focus on morphological variations within species and few studies relate such variation of traits among species. In addition, traits tend to be conserved among closely related species, and therefore, phylogenetic relatedness must be taken into account (Wiens et al. 2010).

A study of insect community structure was carried out on Mount Wilhelm in Papua New Guinea (Leponce et al. 2016). Derbidae, Achilidae, and Cixiidae were the most abundant families among the Fulgoromorpha collected (Le Cesne et al. 2015). Cixiidae are known for being phloem feeders (Wilson et al. 1994). The epigean species feed on the aerial parts of the plants and generally feed on only a few species of plants (Holzinger et al. 2002). Most of the Cixiidae host plants in the tropics remain unknown except for some occasional studies such as Attié et al. (2008) in the Mascarene Islands. In Bourgoin (2025) 473 host plants are listed for this family that contain 2640 described species. Juveniles of Achilidae and Derbidae live under bark and are associated with rotting wood (O'Brien and Wilson 1985; O'Brien 2002). Achilidae presumably feed on fungal hyphae, favoring Polyporales for the juveniles, while the adults are associated with different plants such as Pinaceae or Fagaceae, according to Asche (2015). For this family of around 521 described species, 85 host plants are recorded (Bourgoin 2025). The host plants of Derbidae are even less known, with only 120 host plants recorded for 1723 described species (Bourgoin 2025). A molecular phylogeny was conducted using specimens identified to morpho‐species of the three families, and their distribution was analyzed (Chatelain et al. 2019). Cixiidae inhabiting the same altitude are phylogenetically closer to each other than to Cixiidae inhabiting other altitudes. According to Chatelain et al. (2019), this shows niche conservatism due to environmental filtering. Cixiidae species at the same altitude are therefore more phylogenetically related than species living at different altitudes (Chatelain et al. 2019). In contrast, Derbidae and Achilidae distribution has not shown niche conservatism, but rather an overdispersion due to competition between sister species. Local adaptation would therefore be different for community structure in Cixiidae and in Derbidae and Achilidae (Chatelain et al. 2019). It is indeed possible that an altitudinal filtering effect acts on the distribution of Cixiidae, resulting in morphological variations linked to the altitudinal distribution of the species, but with conserved morphological traits between sister species. Conversely, Derbidae and Achilidae may present a variation of traits along the altitudinal gradient, but not conserved between sister species. The shape and size of the forewings may reflect dispersal capacity, and the length of the rostrum may reflect adaptation to the host plant. Fulgoromorpha are all phytophagous, feeding on their host plants with their modified, rostrum‐forming mouthparts (Cook and Denno 1994). Since the plant community changes with altitude, we expect Fulgoromorpha to display mouthpart morphology adapted to different host plants depending on their altitudinal distribution (Givnish 1999). We may also observe an effect of altitude on the morphology of their forewings in relation to their dispersal capacity. However, these traits should be conserved between closely related species of Cixiidae but not between closely related species of Derbidae and Achilidae.

## 2 Material and Methods

### 2.1 Study Area

Insect sampling was set up along an altitudinal gradient in Papua New Guinea organized by the Muséum National d'Histoire Naturelle and Pro‐Natura International. Sampling was carried out at eight different altitudes every 500 m extending from 200 to 3700 m on Mount Wilhelm (Leponce et al. 2016). The study was conducted on the northeast part of Mount Wilhelm, and the transect followed the crests of the east slope of the mountain from 5°44′14.89″ S, 145°19′56.13″ E to 5°47′27.23″ S, 145°3′29.58″ E. At elevations less than 1000 m, the tropical rainforest is dominated by Dipterocarpaceae, the average daily temperature fluctuates between 25°C and 30°C, and annual rainfall is greater than 4000 mm. Between 1000 and 2500 m, Lauraceae and Fagaceae are dominant, and the average daily temperature ranges from 15°C to 20°C. From 2500 to 3000 m, Podocarpaceae become increasingly abundant, and the average daily temperature is around 12°C. Above 3000 m, the sub‐alpine vegetation is dominated by tree ferns, Cyatheaceae, the average daily temperature is around 8°C, and annual rainfall is less than 3400 mm (Hope 1976; Munzinger et al. 2013).

Specimens were collected by malaise traps during 2 weeks between October and November 2012.

### 2.2 Fulgoromorpha Sampling

Four traps were positioned at each elevation, 100 m apart from each other, totaling 32 collecting points covering the gradient. Distances between elevations vary between 2.5 and 30.4 km. Specimens were collected daily and preserved in 90% ethyl alcohol. Specimens of Fulgoromorpha families of Achilidae, Cixiidae, and Derbidae were sorted, totaling 59 morpho‐species (Le Cesne et al. 2015).

**FIGURE 1:**
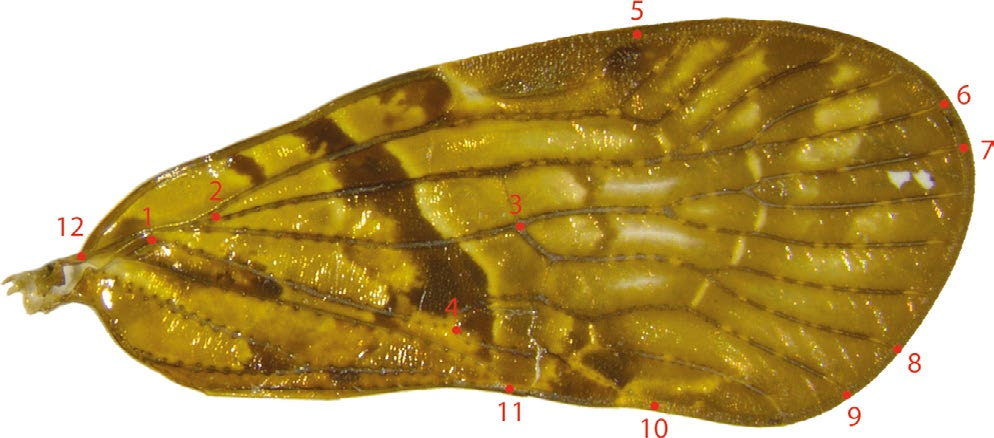
Placement of reference landmarks (points 1–11) and starting landmark for wing contour and length measurement (point 12) on a Cixiidae forewing.

Forewing and rostrum size, as well as forewing shape, were used to analyze morphological adaptation to altitude. Measurements were done on all the morpho‐species, except nine too damaged to be measured. Forewing shape was figured by 11 anatomical landmarks identified and associated with coordinates. Variations in these coordinates were analyzed without taking into account variation due to other factors such as position or size (Adams and Otárola‐Castillo 2013). Anatomical landmarks on forewing photos were placed using tpsDig software. The contour of each forewing was traced and represented by 200 “sliding” points, or sliding semi‐landmarks, placed at regular intervals (Gunz and Mitteroecker 2013). Seven of the selected landmarks are located at the edge of the forewings, and the other four are placed inside the forewings. These landmarks were selected in relation to the forewing venation pattern (Figure 1), and their anatomical significance is described in Table 1. The venation model of Bourgoin et al. (2014) was used to identify each vein. In order to draw the forewing contour, the starting landmark corresponds to the basal end of the forewing.

**TABLE 1:** Landmark significance on the forewing venation.

Landmark number	Anatomical significance
1	Contact point of Cubital Posterior vein and basal cell
2	Bifurcation of Sub‐costal Posterior + Radial + Median Anterior vein in Sub‐costal Posterior + Radia Anterior vein and Radial Posterior + Median Anterior vein
3	First division of Median Posterior vein
4	First division of Cubital Anterior vein
5	Contact point between Sub‐Costal Posterior vein and wing margin
6	Contact point between the most posterior Radial Posterior vein division and wing margin
7	Contact point between the most anterior Median Posterior vein division and wing margin
8	Contact point between the most posterior Median Posterior vein division and wing margin
9	Contact point between the most anterior Cubital Anterior vein division and wing margin
10	Contact point between the most posterior Cubital Anterior vein division and wing margin
11	Contact point between Cubital Posterior vein and wing margin
12	Basal point, for measurement of wing length, placed at the confluence of Costal margin and Sub‐costal + Radial + Median Anterior vein

Forewing, rostrum, and eye lengths were measured from photographs using tpsDig software. Forewing length is taken from the same point as the starting landmark for the forewing outline, point no. 12 (Figure 1), to the distal end of the forewing, so as to measure the maximum length. The length of the rostrum is measured from the distal end of the labrum to the distal end of the rostrum. Eye measurement is taken by measuring the largest diameter and is used to establish forewing/eye length and rostrum/eye length ratios. It is used instead of the total size of the individual, on the assumption that the size of the eyes is a proxy for body size, which could not be measured because the abdomen was missing on several individuals. Abdominal size can also vary between males and females or according to whether the abdomen is more or less dilated. Measuring the eyes has the advantage of measuring an element not disproportionate to body size in these species.

### 2.3 Data Analyses

**FIGURE 2:**
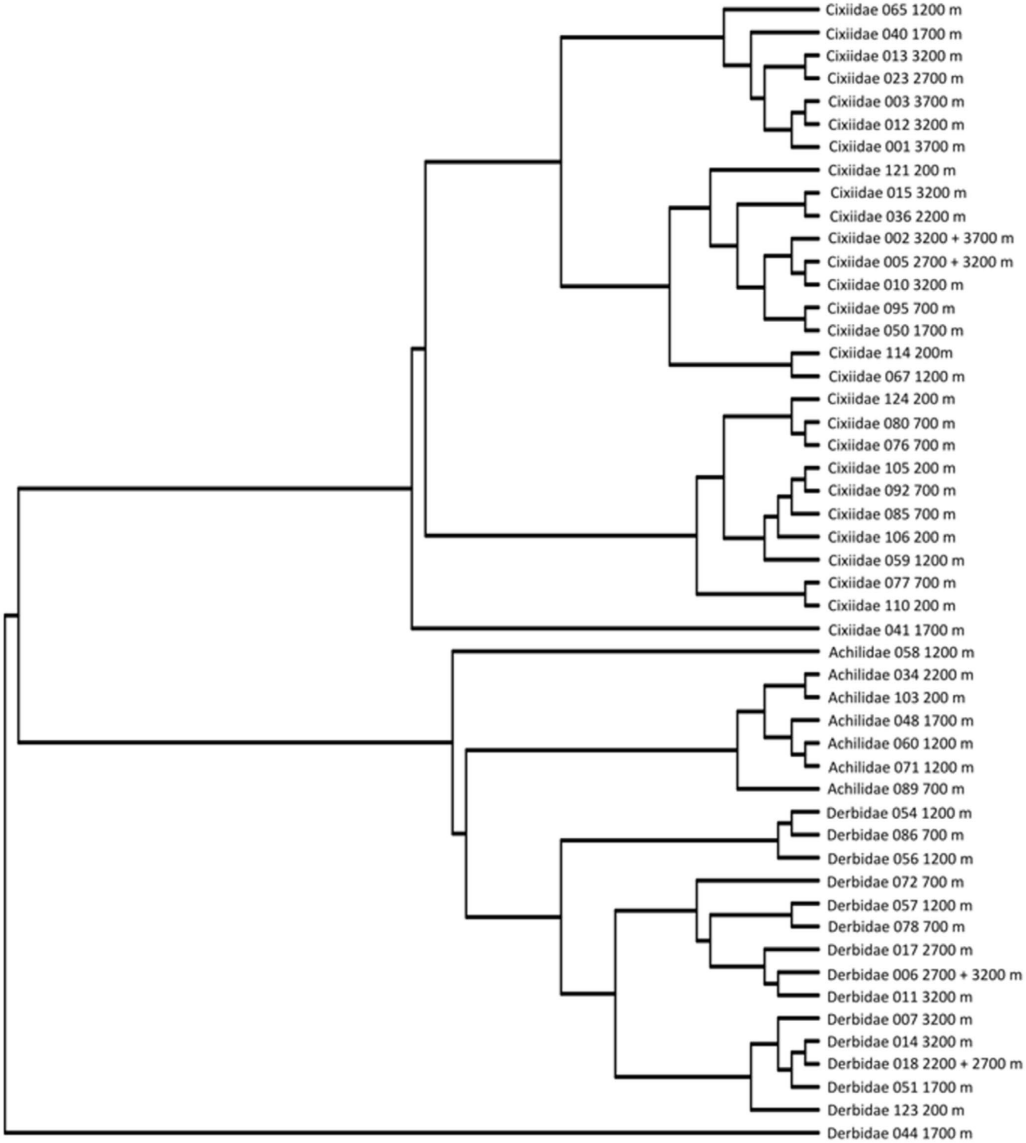
Tree topology showing the two main clades (Cixiidae and Achilidae + Derbidae) with the altitude where each morpho‐species was collected. Number refers to the morpho‐species selected for the analysis.

The phylogenetic tree used to analyze morphological variations included 65 terminals, comprising 59 of the 134 morpho‐species collected along the gradient (Chatelain et al. 2019). It was obtained on the basis of three molecular markers, namely the Cytochrome Oxidase subunit (1658 bp), the ribosomal 18S subunit (1359 bp) and Histone 3 (340 bp), totaling 2357 bp, by Bayesian inferences. The six outgroups were eliminated from the phylogenetic tree for statistical tests on R, as they were outside the group of interest. The tree contains mainly Cixiidae grouped in one clade and Derbidae together with Achilidae in another clade (Figure 2).

Forewing shape was analyzed using ‘Geomorph’ package on R (Adams and Otárola‐Castillo 2012). Procruste superpositions were carried out with ‘gpagen’ function. This involves minimizing the differences in position and size of each specimen and superimposing them in the same coordinate system so that only variations in shape are taken into account (Adams et al. 2013; Rohlf and Slice 1990). A Principal Component Analysis (PCA) was performed on the shape coordinates in order to synthesize the information and identify forewings with similar shapes, using ‘gm.prcomp’ function. PCA was done taking into account phylogenetic distance between species. Graphical functions were used to visualize the variation of shapes along the PCA axes in relation to the mean shape, and to visualize the shape of a particular forewing to observe differences in shape along the first two PCA axes. We calculate phylogenetic diversity using Mean Pairwise Distance (MPD) and Mean Nearest Taxon Distance (MNTD) (Webb et al. 2002). MPD test calculates the average phylogenetic distance between each species at each altitude, and the MNTD test calculates the average distance to the nearest taxon for each species at each altitude (Kembel et al. 2010). MPD is sensitive to changes at distant taxa of the phylogeny; while MNTD is sensitive to changes at the tips of the phylogeny. For each of these indices, the Standardized Effect Size (SES) is calculated by comparing the MPD or MNTD measure observed to the one of a random community, with no particular structure, via the formula: $\mathrm{SES}\;\text{metric}=\text{Metric observed}–\text{mean}\;\left(\text{Metric null}\right)\;\mathrm{sd}\;\left(\text{Metric null}\right)$ (Kembel et al. 2010).

The presence of a phylogenetic signal in morphological traits was detected using an adaptation of the Kappa statistic of Blomberg et al. (2003) to multivariate data (Adams 2014), using ‘physignal’ function and based on 1000 permutations. To determine whether altitude influences morphological traits even when the effect of phylogeny was taken into account, a phylogenetic ANOVA was performed using the ‘procD.pgls’ function.

The presence of a phylogenetic signal on absolute and relative rostrum and forewing lengths was evaluated using ‘multiPhylosignal’ function of ‘ape’ package.

A linear model was applied on the four morphological traits selected to see if any trend in size was related with the altitude, using ‘stats’ package. Normality of residuals was tested with Shapiro test. The four traits were tested for the whole clade and for the two clades representing Cixiidae on one side and Achilidae‐Derbidae on the other side.

## 3 Results

SES values traduce a tree‐wide pattern of phylogenetic clustering at 200 m (SES = −2.772; *p*‐value = 0.04); while they traduce a clustering towards the tips at 3200 and 3700 m (SES = −0.871 and −1.724, respectively; *p*‐values = 0.01 for both). Positive values of SES (1.331) and high *p*‐values (0.980) at 1700 m traduce a phylogenetic evenness (Table 2).

**TABLE 2:** MPD and MNTD tests showing the observed values by altitude, the SES, and *p*‐values for the entire phylogeny including Cixiidae and Achilidae‐Derbidae. Significant *p*‐values are in bold.

Altitude	*n*taxa	MPD	SES	*p*	MNTD	SES	*p*
200	8	25.997	−2.772	**0.040**	24.000	−0.237	0.455
700	12	70.984	−0.154	0.320	14.833	−0.806	0.195
1200	12	73.965	0.222	0.560	15.000	−0.798	0.210
1700	8	69.245	1.331	0.970	46.250	2.632	0.980
2200	4	51.840	−0.601	0.190	47.500	0.033	0.530
2700	5	46.050	−1.019	0.160	24.400	−1.153	0.155
3200	11	60.708	−0.871	0.150	5.636	−2.369	**0.010**
3700	3	19.302	−1.724	0.065	15.333	−2.153	**0.010**

When considering only the clade of Cixiidae, SES values are mostly negative and then traduce a clustering, except at 1700 m. Such a phylogenetic clustering is significant at 200 and 3200 m (SES = −2.245 and −3.275, respectively; *p*‐value = 0.040 and 0.010, respectively; Table 3).

**TABLE 3:** MPD and MNTD tests showing the observed values by altitude, the SES, and *p*‐values for the clade of Cixiidae. Significant *p*‐values are in bold.

Altitude	*n*taxa	MPD	SES	*p*	MNTD	SES	*p*
200	6	13.742	−2245	**0.040**	14.667	−0.536	0.315
700	7	26.222	−1557	0.080	8857	−1238	0.105
1200	5	31.120	−0.166	0.350	14.000	−0.868	0.220
1700	3	18.420	0.668	0.700	45.333	1172	0.925
2200	1	NA	NA	NA	NA	NA	NA
2700	2	14.250	−0.004	0.460	38.000	−0.325	0.340
3200	6	10.270	−3275	**0.010**	6000	−2186	**0.010**
3700	3	19.302	−0.726	0.245	15.333	−1324	0.065

When considering only Achilidae‐Derbidae, SES values are negative between 2200 and 3200 m, but the phylogenetic clustering is significant only at 3200 m (SES = −2.398; *p*‐value = 0.020; Table 4).

**TABLE 4:** MPD and MNTD tests showing the observed values by altitude, the SES, and *p*‐values for the clade including Achilidae and Derbidae. Significant *p*‐values are in bold.

Altitude	*n*taxa	MPD	SES	*p*	MNTD	SES	*p*
200	2	23.111	0.909	0.745	52.000	0.970	0.770
700	5	24.082	−0.789	0.220	23.200	0.401	0.620
1200	7	35.592	0.913	0.830	15.714	0.001	0.505
1700	4	29.000	0.395	0.575	28.500	0.547	0.695
2200	3	22.000	−0.003	0.525	24.000	−0.338	0.480
2700	3	15.642	−1058	0.175	15.333	−1110	0.170
3200	5	11.680	−2398	**0.020**	5200	−1994	**0.010**
3700	0	NA	NA	NA	NA	NA	NA

When pulling together altitudes in three vegetation strata, SES values traduce a phylogenetic clustering at lower altitudes and at higher altitudes (MPD SES = −3.580 and MNTD SES = −0.800, respectively; *p*‐values = 0.01 for both). SES values argue more for a phylogenetic evenness rather than clustering at middle altitudes; however, not significantly (Table 5).

**TABLE 5:** MPD and MNTD tests showing the observed values by vegetation strata, the SES and *p*‐values for the entire phylogeny including Cixiidae and Achilidae–Derbidae. Significant *p*‐values are in bold.

	*n*taxa	MPD	SES	*p*	MNTD	SES	*p*
low	18	38.861	−3580	**0.010**	11.556	−0.834	0.215
middle	18	77.822	1061	0.890	24.000	2060	0.950
high	15	65.447	−0.800	0.160	4133	−2511	**0.010**

Phylogenetic clustering is significant at lower and higher altitudes for the Cixiidae considered separately (MPD SES = −3.579 and −2.734, respectively; *p*‐value = 0.010 and 0.030, respectively; Table 6). Phylogenetic evenness is significant at middle altitude with MNTD test (SES = 2.243; *p*‐value = 0.995).

**TABLE 6:** MPD and MNTD tests showing the observed values by strata, the SES and *p*‐values for the clade of Cixiidae. Significant *p*‐values are in bold.

	*n*taxa	MPD	SES	*p*	MNTD	SES	*p*
low	12	15.084	−3579	**0.010**	6667	−1315	0.105
middle	7	31.520	0.297	0.600	27.429	2243	0.995
high	9	18.355	−2734	**0.030**	3556	−2335	**0.010**

There is a significant phylogenetic clustering at higher altitude also for Achilidae‐Derbidae taken separately with MNTD test (SES = −2.072; *p*‐value = 0.020; Table 7). Such a clustering is almost significant also with MPD test (SES = −2139; *p*‐value = 0.05).

**TABLE 7:** MPD and MNTD tests showing the observed values by strata, the SES and *p*‐values for the clade of Achilidae‐Derbidae. Significant *p*‐values are in bold.

	*n*taxa	MPD	SES	*p*	MNTD	SES	*p*
low	6	33.813	0.524	0.750	21.333	0.642	0.755
middle	10	36.860	1190	0.890	12.000	−0.094	0.485
high	6	15.207	−2139	**0.050**	5000	−2072	**0.020**

**FIGURE 3:**
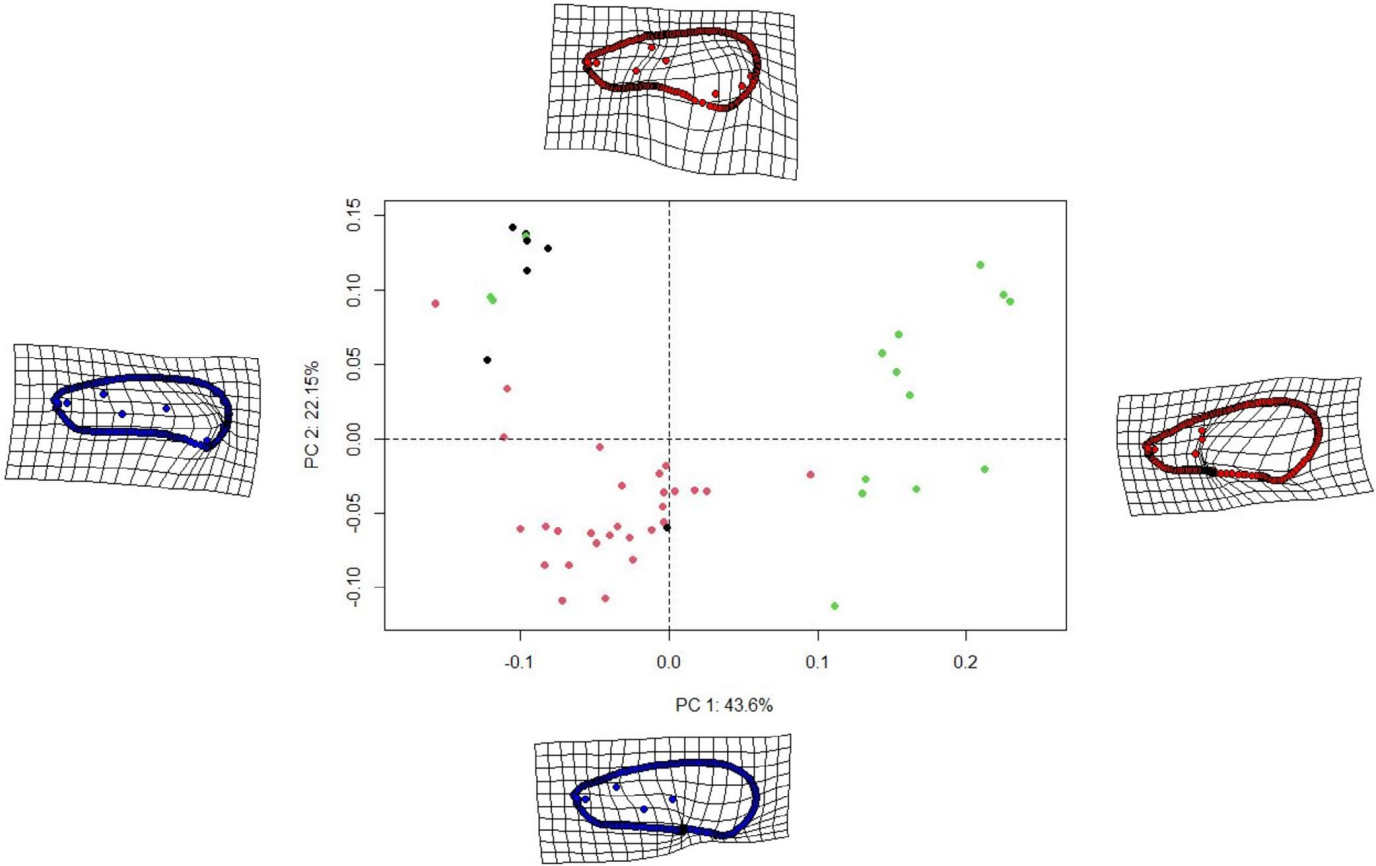
PCA plot of forewing shape data. Cixiidae are shown in red, Derbidae in green and Achilidae in black. Shape variations along each axis are shown at the end of the corresponding axis.

The first two axes of the PCA on forewing shapes explain respectively 43.6% and 22.15% of the variance (Figure 3). The graph showing the distribution of individuals along the axes shows a grouping of most of the Cixiidae together at the left of the first axis, most of the Derbidae together at the right side of the first axis, and Achilidae together at the left side of the first axis and the upper side of the second axis, with a few deviating individuals on the first two axes (Figure 3).

Forewing shape shows a significant phylogenetic signal (*K* = 0.254; *p*‐value = 0.001; effect size = 4.729) but relatively weak since *K* is close to 0. The phylogenetic ANOVA shows a significant effect of altitude on forewing shapes (Table 8). The trait diverges less quickly than in a Brownian model; that would translate to a conservatism in the shape of the forewings.

**TABLE 8:** Results of phylogenetic ANOVA on the effect of altitude on forewing shape (DF, degrees of freedom; SS, sum of squares; MS, mean square; *R* ^2^, *R* squared; *F*, *F* ratio; *Z*, Effect size; Pr, *p*‐value).

	Df	SS	MS	*R* ^2^	*F*	*Z*	Pr(>*F*)
Altitudes	7	0.037	0.005	0.305	2.627	3.941	**0.01**
Residuals	42	0.085	0.002	0.696			
Total	49	0.122					

The K statistic shows a weak but significant phylogenetic signal on absolute and relative forewing length (*K* = 0.091 and 0.122; *p*‐value = 0.030 and 0.001, respectively). There is no significant signal of absolute and relative rostrum length (*K* = 0.084 and 0.089; *p*‐value = 0.102 and 0.1114, respectively). Forewing length is not distributed at random in the phylogeny, while rostrum length is.

When considering Cixiidae separately, *K* statistics are not significant for all the traits. All traits show a phylogenetic signal, except the relative length of the rostrum when considering Achilidae–Derbidae separately (Table 9).

**TABLE 9:** K statistic and *p*‐values of the four traits along the altitudinal gradient, considering Cixiidae and Achilidae‐Derbidae separately. Bold numbers represent a significant phylogenetic signal.

Traits	*K* Cixiidae	*p* Cixiidae	*K* Achilidae‐Derbidae	*p* Achilidae‐Derbidae
Forewing	0.108	0.532	0.275	**0.033**
Rostrum	0.086	0.812	0.268	**0.040**
Forewing/eye	0.100	0.680	0.428	**0.009**
Rostrum/eye	0.097	0.629	0.147	0.515

Linear regressions on the four traits show no relationships between relative and absolute rostrum length with altitude. Conversely, relative and absolute forewing lengths show a positive correlation with altitude, indicating that forewing is larger at high altitude. Residuals show a homogeneous variance, and homoscedasticity of residuals is respected (Table 10).

**TABLE 10:** Statistics of linear regression between the four traits as predictive variables and the altitude as explicative variable, and Shapiro test of normality. Significant *p*‐values are in bold.

	Shapiro test		Linear model				
	*W*	*p*	Intercept	Altitude	*R* ^2^	*F*‐stat	*p*
Forewing	0.9652	0.2037	4.3889	3.5e‐04	0.1243	6.53	**0.0140**
Forewing/eye	0.9821	0.6669	10.69	1.088e‐03	0.2502	15.35	**0.0003**
Rostrum	0.9765	0.4418	0.9594	−3275e‐05	0.0092	0.4249	0.5177
Rostrum/eye	0.9867	0.8559	2.313	−7.297e‐05	0.0129	0.6001	0.4425

When considering Cixiidae and Achilidae‐Derbidae independently, the linear regressions show a significant correlation between both clades with the altitude for the absolute and relative forewing length. The correlation is more significant for the Achilidae‐Derbidae than for the Cixiidae. Linear regressions also show a significant correlation between absolute and relative rostrum length for Achilidae‐Derbidae, but not for Cixiidae (Table 11).

**TABLE 11:** Linear regressions of the four traits as predictive variables and the altitude as explicative variable and Shapiro test of normality for the Cixiidae (C) and the Achilidae‐Derbidae (A + D) separately. Significant *p*‐values are in bold.

	Shapiro test		Linear model					
	*W*	*p*	Intercept	Altitude	Pr(<|*t*|)	*R* ^2^	*F*‐stat	*p*
Forewing A + D	0.9686	0.2233	4.3776	0.0003924	*	0.127	3.261	**0.0476**
Forewing C				0.0003347	*			
Forewing/eye A + D	0.9381	**0.0137**	10.39	2.146e‐03	***	0.595	33.12	**1.436e‐09**
Forewing/eye C				6.055e‐04	**			
Rostrum A + D	0.9796	0.5614	1.002	−1.818e‐04	**	0.286	9.001	**0.0005**
Rostrum C				3.522e‐05				
Rostrum/eye A + D	0.9758	0.4176	2.378	−2.985e‐04	*	0.192	5.354	**0.0082**
Rostrum/eye C				2.984e‐05				

## 4 Discussion

A clustering was found for the whole group and for Cixiidae in Chatelain et al. (2019), using other metrics: Ist, Pst, P*st, and Πst (see Hardy 2008 for description). Our results are more contrasted since the clustering is located at 200, 3200, and 3700 m. In addition, it concerns not only Cixiidae but also Achilidae‐Derbidae at 3200 m. They traduce a niche conservatism, mainly in Cixiidae and at high altitudes. The clustering at 200 m concerns Cixiidae and only the MPD value, not MNTD. MPD is more sensitive to distant taxa, and therefore does not traduce conservatism of niche among nearest species.

Cixiidae and Achilidae‐Derbidae have different forewing shapes, and these shapes are phylogenetically conserved. The absence of phylogenetic signal for forewing length and absolute rostrum length in Cixiidae traduces a morphological conservatism congruent with the niche conservatism observed. As niche conservatism is mainly observed at high altitudes, trait conservatism could be due to an adaptation to high altitudes where environmental conditions are more contrasting. Atmospheric pressure, for example, acts as a strong filter as it requires higher flight performance (Hodkinson 2005). Dudley (2002) and Dillon et al. (2018) also suggest wing size increases with altitude to maintain flight performance at lower air pressure. Temperature decreases and wind increases with high elevation, requiring better flight performance of species inhabiting. Conversely, the phylogenetic signal observed in Achilidae‐Derbidae traduces a better capability to disperse along the elevational gradient. Forewing size increases with altitude for Achilidae‐Derbidae and for Cixiidae; however, more significantly for Achilidae‐Derbidae. Note that no Achilidae or Derbidae were found at 3700 m, the highest altitude. Such an absence could be due to dispersal capability to escape too contrasting environmental conditions. High elevation may restrict the presence of less tolerant species to difficult climatic conditions, and this could be the case for Achilidae and Derbidae.

The rostrum size also increases with altitude for Achilidae‐Derbidae but not for Cixiidae. Such a trait could be related to host plant adaptation as trophic preferences are not the same for Cixiidae compared to Achilidae and Derbidae. However, host plants are unknown for these species. No study relates to rostrum length as Hemiptera are poorly studied in such a frame. These first results on such a trait suggest that host plants may count as an environmental filter in the adaptation of species to high altitude, as far as rostrum length is a good proxy for host plant specificity in Hemiptera (Favret and Voegtlin 2004).

These trends have also been observed in other insect groups. Wing size decreases with altitude in Lepidoptera assemblages in a temperate altitudinal gradient (Leingärtner et al. 2014) and also in bees in the Alps (Peters et al. 2016). Conversely, in tropical mountains, wings of butterflies tend to be larger at high altitudes also, and such a trait tends to be phylogenetically conserved (Henriques et al. 2022). Wings tend to be larger for *Heliconius* species at high altitudes (Montejo‐Kovacevich et al. 2019). The shape of the wing differs also according to the altitude in butterflies as well (Montejo‐Kovacevich et al. 2019). In the present case, forewing length and shape of Fulgoromorpha tend to increase with altitude. It confirms the rule that wing size increases and wing shape changes with altitude, despite differences in evolutionary pathways.

Our findings are congruent with other results highlighting the environmental filtering occurring at high altitudes in tropical mountains. Such studies question the effect of climatic changes on community structure and species ecological niche as temperature and other abiotic conditions vary with the altitude. If wing shape and size are quite often used as a proxy of flight capability, the rostrum is rarely studied as a proxy of trophic preferences for phytophagous species related to altitudinal gradients.

## Author Contributions

**De Filippo Elsa:** formal analysis (equal), investigation (equal), methodology (equal), software (equal), validation (equal), visualization (equal), writing – original draft (equal), writing – review and editing (equal). **Soulier‐Perkins Adeline:** conceptualization (equal), data curation (equal), formal analysis (equal), investigation (equal), methodology (equal), supervision (equal), validation (equal), visualization (equal), writing – original draft (equal), writing – review and editing (equal). **Cornette Raphaël:** conceptualization (equal), formal analysis (equal), investigation (equal), methodology (equal), supervision (equal), validation (equal), visualization (equal), writing – original draft (equal), writing – review and editing (equal). **Guilbert Eric:** conceptualization (equal), data curation (equal), formal analysis (equal), methodology (equal), supervision (equal), validation (equal), writing – original draft (equal), writing – review and editing (equal).

## Conflicts of Interest

The corresponding author declares on behalf of all authors that they have no competing or conflicting interest.

## Data Availability

Morphometric and morphologic measurements of specimens used in this study are accessible at https://doi.org/10.5061/dryad.0zpc8676f.
